# Editorial

**DOI:** 10.1016/j.stemcr.2024.102390

**Published:** 2025-01-14

**Authors:** Janet Rossant

**Affiliations:** 1 Editor in Chief

## Main text

**Figure:**
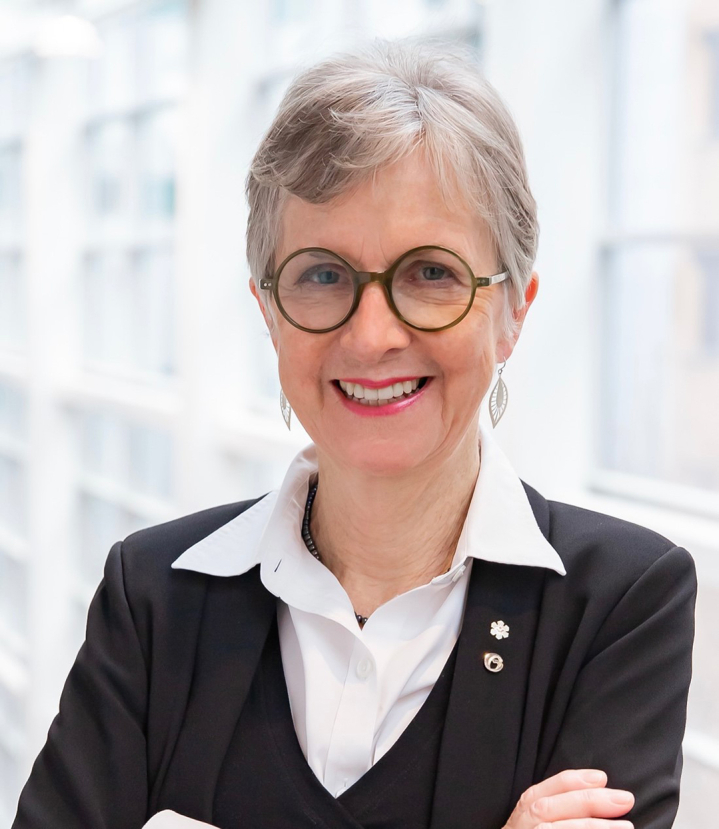
Janet Rossant, PhD, Editor-in-Chief of Stem Cell Reports

I am excited to take on this important role as Editor-in-Chief for Stem Cell Reports, the official journal of the International Society for Stem Cell Research (ISSCR). I have watched with pride the establishment and the ongoing growth of the journal in terms of its areas of focus and its impact over the past few years. I hope to build on this strong foundation and make sure the journal continues to attract new readers to the outstanding science submitted by our community of stem cell researchers, within and outside of ISSCR.

Stem Cell Reports is a leading peer-reviewed journal dedicated to publishing high-quality research and insights in the field of stem cell biology. The journal serves as a vital platform for scientists, clinicians, and industry professionals to disseminate their findings on stem cell research, regenerative medicine, and cellular therapies. Its values reflect those of its host society, ISSCR: trustworthy and rigorous science as judged by scientific editors and reviewers who respect the standards laid down by ISSCR. Stem Cell Reports aims to be the trusted voice for stem cell science globally.

I aim to ensure that the journal truly represents the broad scope of stem cell science, from fundamental developmental biology of stem/progenitor cells in model organisms right through to the results of ongoing clinical trials with adult stem cells and progenitors and differentiated cells derived from pluripotent stem cells. Using stem cells to model human development and disease in novel ways through organoids, embryo models, and microfluidic devices are just a few of exciting areas where we hope to see more papers in the next years.

The stem cell field as a whole is moving rapidly from discovery to application to human health. I hope to see more preclinical studies designed to fill the gap between lab models and the first clinical trial in humans, as well as the results of clinical trials themselves. There are lots of regulatory, practical, and clinical challenges along the way. The journal should also be a place for groups to report honestly on such challenges in an open science, sharing mode so that all can learn from each other and accelerate progress.

Technologies are also moving rapidly—single-cell analysis, spatial transcriptomics, barcode lineage tracing, CRISPR genetic screens, and other high-throughput screening modes, all enhanced by the tools of AI. The journal will publish novel technology applications to enhance stem cell science and resource articles documenting large-scale datasets relevant to the field. We also aim to partner with other journals where initial large-scale studies, like the Human Cell Atlas, are published to provide a home for the more focused follow-up studies that are relevant to our readers.

In addition to showcasing groundbreaking research, Stem Cell Reports will continue to encourage discussions and debates on ethical considerations and the societal implications of stem cell science.

But at its heart the journal is about the best of stem cell science and its applications. We invite everyone to submit their best work to be judged by active stem cell researchers who understand the field and want to help you produce and publish impactful science.

I look forward to working with the existing editors and editorial board. They have been diligent in their efforts to ensure that the research published in the journal is of the highest standards and rigor. And, of course, I am delighted that Martin Pera who has guided the journal so well for the past 6 years is going to stay on an active editor in the pluripotency domain.

I want to hear from the broader stem cell community, especially trainees and early career investigators, on how the journal can do more to enhance the experience of publishing with us. We want to make Stem Cell Reports the journal of choice for the best in stem cell science and clinical translation.

